# Climate, human behaviour or environment: individual-based modelling of *Campylobacter* seasonality and strategies to reduce disease burden

**DOI:** 10.1186/s12967-019-1781-y

**Published:** 2019-01-21

**Authors:** Stephen P. Rushton, Roy A. Sanderson, Peter J. Diggle, Mark D. F. Shirley, Alasdair P. Blain, Iain Lake, James A. Maas, William D. K. Reid, Jo Hardstaff, Nicola Williams, Natalia R. Jones, Daniel Rigby, Norval J. C. Strachan, Ken J. Forbes, Paul R. Hunter, Thomas J. Humphrey, Sarah J. O’Brien

**Affiliations:** 1 Modelling, Evidence and Policy Research Group, School of Natural and Environmental Science, Newcastle University, Newcastle upon Tyne, NE1 7RU UK; 2 Lancaster Medical School, Lancaster University, Lancaster, LA1 4YG UK; 3 School of Environmental Sciences, University of East Anglia, Norwich, NR4 7TJ UK; 4 Norwich Medical School, University of East Anglia, Norwich 33, NR4 7TJ UK; 5 Institute of Psychology, Health and Society, University of Liverpool, Liverpool, L69 3BX UK; 6 Institute of Infection and Global Health, Liverpool University, Liverpool, L69 7BE UK; 7 School of Social Sciences, The University of Manchester, Manchester, M13 9PL UK; 8 School of Natural and Computing Sciences/Food Standards Agency Scotland, University of Aberdeen, Aberdeen, AB24 3FX UK; 9 School of Medicine, Medical Sciences and Nutrition, University of Aberdeen, Aberdeen, AB25 2ZD UK; 10 NIHR Health Protection Research Unit in Gastrointestinal Infections, University of Liverpool, Liverpool, UK; 11 School of Medicine, Swansea University, Swansea, SA2 8PP UK; 12 Ecology Research Group, School of Natural and Environmental Science, Newcastle University, Newcastle upon Tyne, NE1 7RU UK

**Keywords:** *Campylobacter*, Individual-based modelling, Risk behaviours, Food, Weather, Vaccination

## Abstract

**Background:**

With over 800 million cases globally, campylobacteriosis is a major cause of food borne disease. In temperate climates incidence is highly seasonal but the underlying mechanisms are poorly understood, making human disease control difficult. We hypothesised that observed disease patterns reflect complex interactions between weather, patterns of human risk behaviour, immune status and level of food contamination. Only by understanding these can we find effective interventions.

**Methods:**

We analysed trends in human *Campylobacter* cases in NE England from 2004 to 2009, investigating the associations between different risk factors and disease using time-series models. We then developed an individual-based (IB) model of risk behaviour, human immunological responses to infection and environmental contamination driven by weather and land use. We parameterised the IB model for NE England and compared outputs to observed numbers of reported cases each month in the population in 2004–2009. Finally, we used it to investigate different community level disease reduction strategies.

**Results:**

Risk behaviours like countryside visits (t = 3.665, P < 0.001 and t = − 2.187, P = 0.029 for temperature and rainfall respectively), and consumption of barbecued food were strongly associated with weather, (t = 3.219, P = 0.002 and t = 2.015, P = 0.045 for weekly average temperature and average maximum temperature respectively) and also rain (t = 2.254, P = 0.02527). This suggests that the effect of weather was indirect, acting through changes in risk behaviour. The seasonal pattern of cases predicted by the IB model was significantly related to observed patterns (r = 0.72, P < 0.001) indicating that simulating risk behaviour could produce the observed seasonal patterns of cases. A vaccination strategy providing short-term immunity was more effective than educational interventions to modify human risk behaviour. Extending immunity to 1 year from 20 days reduced disease burden by an order of magnitude (from 2412–2414 to 203–309 cases per 50,000 person-years).

**Conclusions:**

This is the first interdisciplinary study to integrate environment, risk behaviour, socio-demographics and immunology to model *Campylobacter* infection, including pathways to mitigation. We conclude that vaccination is likely to be the best route for intervening against campylobacteriosis despite the technical problems associated with understanding both the underlying human immunology and genetic variation in the pathogen, and the likely cost of vaccine development.

## Background

*Campylobacter* species are the most important gut pathogens in developed countries. Campylobacteriosis occurs in 1% of the US population each year [1] and costs the European Union alone an estimated €2.4 billion annually [2]. In developing countries the disease is endemic but extensively unrecorded and it is prevalent in infants (< 1 year), with isolation rates of 8 to 21% of all diarrhoea samples [3]. In developed countries the disease also occurs in older age groups. There is considerable pressure to reduce disease burden with government agencies having strategies to monitor disease. The public health burden, however, continues to rise. Illness is often associated with consumption of chicken [4–9] but this does not account for all cases [10]. In temperate regions *Campylobacter* incidence is also predictably seasonal [10, 11] but the causes of this seasonality are not understood. *Campylobacter* is found in many animal species and these along with environmental exposures have been suggested to explain 20–40% of disease burden [12]. The relative importance of different exposures to disease remains largely unquantified which renders effective intervention to reduce the disease burden difficult. Furthermore, understanding of the interaction between human host and pathogen is poor as seroconversion rates are variable (67–96%) and infections can be asymptomatic [13]. There is also a dose–response relationship for infection [14, 15], but not symptoms [16].

Why is the disease seasonal in developed countries? Understanding the causes of seasonality could help identify methods for mitigating against disease when it is most prevalent. Exposure to *Campylobacter* is multifactorial, in that the pathogen is probably ubiquitous in the environment and in much raw chicken. To understand how the disease spreads requires understanding of human risk behaviours, social demography; consideration of how contact with the pathogen comes about and how it leads to disease. In effect we need to integrate across a range of ‘epidemiological’ processes that operate at different scales. Here we use an interdisciplinary approach to investigate different pathways of exposure to *Campylobacter* strains via the rural environment and diets, and link these to potential seasonality in human risk-behaviours. We then attempt to determine the most effective interventions to mitigate disease. We used a combined biostatistical and individual-based (IB) modelling approach. We used time-series analyses to investigate the role of weather in disease and in mediating those human risk-behaviours that increase exposure to the pathogen and hence disease. We sought to identify the extent to which disease is related to weather after adjusting for seasonality numerically for a real population where the disease burden was known. One issue with analysing data that show seasonality is that apparent associations may occur between two or more variables, but the correlation does not reflect a causal link between the variable as there is another (often unmeasured variable) driving both processes. We used harmonic regression to model the relationship between the pattern of cases and human risk behaviours and month. This approach allowed us to adjust for seasonality and identify the direct and indirect roles of weather that determine exposure to *Campylobacter* associated with eating chicken, cooking activities and countryside visits. However, this approach did not allow us to quantify the relative importance of each risk behaviour in causing disease, a key outcome if we are to identify methods to intervene to mitigate against disease. To evaluate the contribution of these different exposure pathways to disease we developed an IB model which models stochastically the daily experience of human individuals, their risk-behaviours and immunity, and integrates with weather and exposure, to predict disease. We tested this model by predicting temporal disease patterns in a large population of individuals in North East England, UK. The region has a population of 910,000 with an area in excess of 2500 km^2^, at 55° latitude N. Finally, we used the IB model to investigate how interventions to extend the duration of immunity and to reduce risk behaviours might reduce the burden of disease.

## Methods

### Time-series analyses of cases of disease, human risk behaviours and weather

We investigated the effects of seasonality in temperature and rainfall on three human risk behaviours: visits to the countryside, potential barbecue activity and purchase of chicken products for barbecue. Completely coterminous data were not available for all variables, so we assumed that patterns observed over all periods were consistent: it is well-established that seasonality in cases is consistent over long periods of time in the UK [17].

### Data collation

Monthly occurrence of *Campylobacter* cases, daily temperature and rainfall from 2005 to 2009 and 2010 to 2015 were collated for NE England [18]. Visits to the countryside by the public were obtained from the Monitor of Engagement with the Natural Environment survey (MENE) [19] based on interviews of 800+ participants/week across NE England from 2009 to 2015. A proxy variable of barbecue activity in the region was constructed from the internet queries per month for barbecue charcoal in England on Google Trends from 2012 to 2015. Weekly sales of all fresh chicken products were obtained for 2013 to 2015 from one of the UK’s largest UK supermarkets.

### Time-series analyses

To investigate the relationships between *Campylobacter* cases, weather and the three risk-behaviours we de-seasonalised each variable using six sine-cosine harmonic regressions [18]:

- Mean temperature/month,
- Total rainfall/month,
- *Campylobacter* cases/month,
- Barbecue charcoal queries/month,
- Sales of broiler chicken/month,
- Number of visits to the countryside/day,
- Sales of barbecue chicken/month.

Temperature, rainfall, *Campylobacter* cases, charcoal queries and chicken sales were de-seasonalised with an annual cycle whereas visits to the countryside, where more fine-grained data were available, were de-seasonalised for weekly and annual periods.

The residuals of each temporal model were used as de-seasonalised representations of the original response variable. Linear regressions were used to determine the relationships between de-seasonalised temperature and rainfall (independent weather predictors) versus de-seasonalised *Campylobacter* cases and the three risk-behaviours (dependent variables). Likewise de-seasonalised broiler chicken and barbecue chicken sales were compared with de-seasonalised *Campylobacter* cases. Non-significant relationships between a de-seasonalised predictor and a de-seasonalised dependent variable were assumed to indicate that the two variables were independent of each other.

### IB model of impacts of risk behaviours, exposure and immunology on Campylobacter disease

**Fig. 1:**
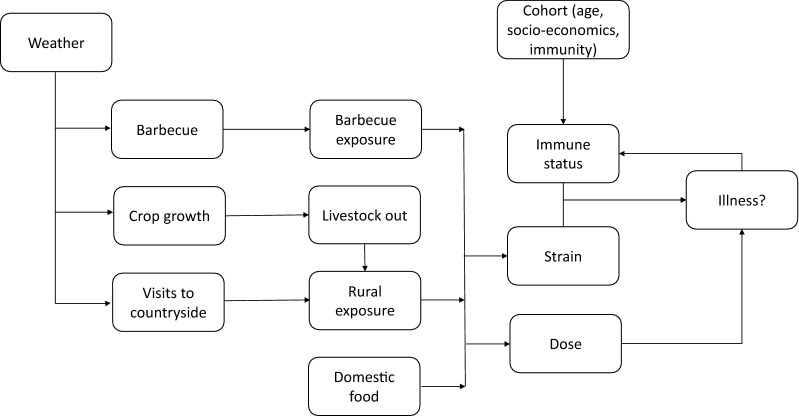
Flow diagram of the IB modelling model

The IB model simulates temporal patterns of risk-behaviours, exposure pathways, immune response, and subsequent probability of disease in relation to seasonal variation in weather, age and socio-economic status for individuals (Fig. 1). The processes considered were:

- Consumption of barbecued food as a source of *Campylobacter*,
- Infection from chicken preparation and consumption in the home,
- Presence of *Campylobacter* in the countryside as determined by livestock land use,
- Visits to the countryside as determined by weather, day of week, age and socio-economic status,
- Human exposure to *Campylobacter* in the countryside,
- Strains encountered when individuals were exposed,
- Immune response of an individual after exposure to *Campylobacter*.

Parameters used in the IB model are summarized in Appendix Table 1.

**Table 1:** Sources of data used for parameterisation of microsimulation model; reference numbers refer to main text

Parameter	Range or type of values	Source
a. Consumption of barbecued food		
Barbecue occurrence	0 to 100	Derived from time-series analyses
Frequency of barbecues per day and across year	Probability on given day of week 0–1 and overall frequency	Idealo Survey 2017 [19]
Contamination of chicken meat	Probability 0–1	Food Standards Agency 2014 [23]
Undercooking of barbecue food	Probability 0–1	Food Standards Agency 2014 [20]
Population that consumes chicken	Proportion 0–1	Poultry Site 2018 [21]
b. Infection from chicken preparation and cooking at home		
Purchased chicken is contaminated	Probability 0–1	Food Standards Agency 2015 [22]
Chicken sold that is skin	Proportion 0–1	Food Standards Agency 2017 [23]
Frequency distribution of *Campylobacter* on chicken skin	Observed frequency distribution	Nauta, Jacobs-Reitsma [24]
c. Presence of *Campylobacter* in the countryside		
Herbage biomass sufficient for 10 days grazing by cows at 2.4 per ha	Gompertz modified biomass growth model	Barker et al. [25]
d. Visits to the countryside		
GEE based on temperature, rainfall, day of week, age, socio-economic class	Probability 0–1	MENE data [18] plus temperature, rainfall and day of week
e. Exposure to *Campylobacter* in the countryside		
Pathogen strain-type frequency distribution	Observed frequency distribution	Jones, Millman et al. [27]
Campylobacter counts in sheep, cattle and wild bird faeces	Observed counts	Stanley, Wallace et al. [28]
Transmission from footware to hands	Probability 0–1	Nauta, Jacobs-Reitsma [24]
f. Immune response after exposure to *Campylobacter*		
Human dose–response experiments	Dose–response curves	Black Levine et al. [13]
Reduction in CFU after barbecue cooking	2.5× reduction	Food Standards Agency 2015 [22]

- Consumption of barbecued food as a source of *Campylobacter*.
  The relationship between charcoal queries and weather, from the time-series analysis (above), was used to predict barbecue occurrence on a scale of 0 to 100. Idealo Survey data [20] were used to quantify the frequency of barbecues and their distribution across the days of the week. Frequency of barbecue was assigned to each individual and also the probability that they would have a barbecue on a specific day of the week. *Campylobacter* exposure was then predicted as the product of two probabilities: first that meat was contaminated [21] and second that the meat was undercooked [21].
- Infection from chicken preparation and consumption in the home.
  We estimated daily consumption of chicken based on the population known to consume this meat [22] and amount of chicken consumed. Surface contamination was calculated from: the probability that a purchased chicken was contaminated [23]; the proportion of the chicken that was skin [24]; and the frequency distribution of *Campylobacter* found on chicken skin purchased from UK retailers [23]. This procedure could not distinguish between barbecue cooking and other forms of chicken consumption, so may have led to an over-estimate of the contribution of chicken. Exposure to cross-contamination and likely transmission were modelled after Nauta et al. [25].
- Presence of *Campylobacter* in the countryside.
  *Campylobacter* strains in the countryside were predicted to arise from sheep, wild birds and cattle. Sheep and wild bird contamination was assumed to be constant throughout the year, whilst that of bovine contamination was seasonal, occurring only after grass growth was sufficient to maintain stock for 10 days. We predicted grass growth using a modified Gompertz model [26]:
  $$y_{t} = a_{1} + \left( {a_{2} - a_{1} } \right)e^{{ - be^{ - ct} }}$$
  where: *y*_*t*_ = herbage biomass after *t* day-degrees; *a*_*1*_*, a*_*2*_*, b, c* = estimated model parameters.
  Scale parameters *a*_*1*_ and *a*_*2*_ were determined by the minimum and maximum values respectively of herbage biomass typical in UK farms. Pastures were predicted to be contaminated by bovine sources if the increase in herbage mass was sufficient to support 10 days of consumption by cows at an average stocking density of 2.4 cows ha^−1^.
- Visits to the countryside.
  Generalized estimating equations (GEE with Wald tests) [27] were used to predict the probability that an individual would visit the countryside from the MENE data. We modelled visit on each day of the week as a logistic response and included an autoregressive correlation structure to account for serial dependency between days using temperature, rainfall, day of the week, age and socioeconomic class as predictors.
- Exposure to *Campylobacter* in the countryside.
  Exposure to *Campylobacter* was assumed to be via footwear. We assumed that on handling foot-ware *Campylobacter* would be transmitted to hands and the relationships of Nauta et al. [25] were used to model the transmission of *Campylobacter* to hands and food.
- Strains encountered when individuals were exposed.
  Pathogen strain-type was derived from the frequency distribution of strain-types recorded in the field [28]. The dose was set arbitrarily at 0.1 g to provide an invisible and conservative estimate of contamination. *Campylobacter* counts in sheep, cattle and wild bird faeces were derived from Stanley et al. [29].
- Immune response of an individual after exposure to *Campylobacter.*
  We assumed that the dose consumed affected the likelihood of becoming ill [30]. Exposure may or may not result in illness [14, 15] but only cases with moderate or severe illness will be reported. Illness depends on both dose [14, 15] and extent of previous exposure and immunity. We modelled the illness response of humans to exposure using data derived from human dose response experiments [14] and assumed that cooking on a barbecue would result in a 2.5-fold reduction in the dose of colony forming units (CFU) [23]. The modelled dose was used to predict the likelihood of illness subject to the predicted level of immunity at the time of exposure. Immunity was assumed to decline exponentially from time of exposure to zero at a pre-defined time, which could be set as a model input variable. Whilst exposure to *Campylobacter* may not cause illness, the antigens present may still initiate a response from the host immune system, so any exposure to *Campylobacter* which did not lead to illness was assumed to affect immunity and return it to 100% as would occur immediately after illness. We did not simulate different immune responses for different strains.

### Validation of the IB model

The model was run for NE England using weather data from January 2005 to November 2009. A cohort population of 10,000 individuals was created for each simulation. Individuals were assigned age, gender and socio-economic class based on the socio-economic structure in NE England. The initial immune status of individuals was a normal random deviate (mean 0.5, SD 0.2). We predicted cases for the whole population and compared with the log-transformed monthly number of cases using generalized linear models (GLM). We ran the model 10 times from the same starting conditions and produced a mean number of cases per month and associated standard errors on our predictions.

### Modifying human risk behaviours and immunity to mitigate against disease

We varied parameter estimates for risk behaviours, weather and immunity.

The following input parameters were used:

- Extending the period of immunity leading to protection from developing disease (21 to 1095 days) as might be undertaken following an intervention to enhance immunity following infection, such as vaccination with a hypothetical polysaccharide vaccine that produced short-term immunity.
- Probability of chicken being undercooked (contamination reduction per cooking event) as would occur following implementation of an education program to reduce risk of exposure in domestic settings.
- Fold-reduction in CFU dose in food from either cooking or reducing the burden in raw chicken (1.5 to 2.5) as would occur following implementation of an education program or a scheme to reduce the cfu on raw chicken during production.
- Temperature (± 2.5 °C) and rainfall (± 10 mm). These assess impacts of weather on visits to the countryside and barbecue behaviour.

We used Latin Hypercube Sampling [31] to create ranges for input parameters and used GLMs to quantify the contribution of each variable to the predicted number of cases.

## Results

### Impacts of temperature and rainfall on *Campylobacter* cases in NE England

**Fig. 2:**
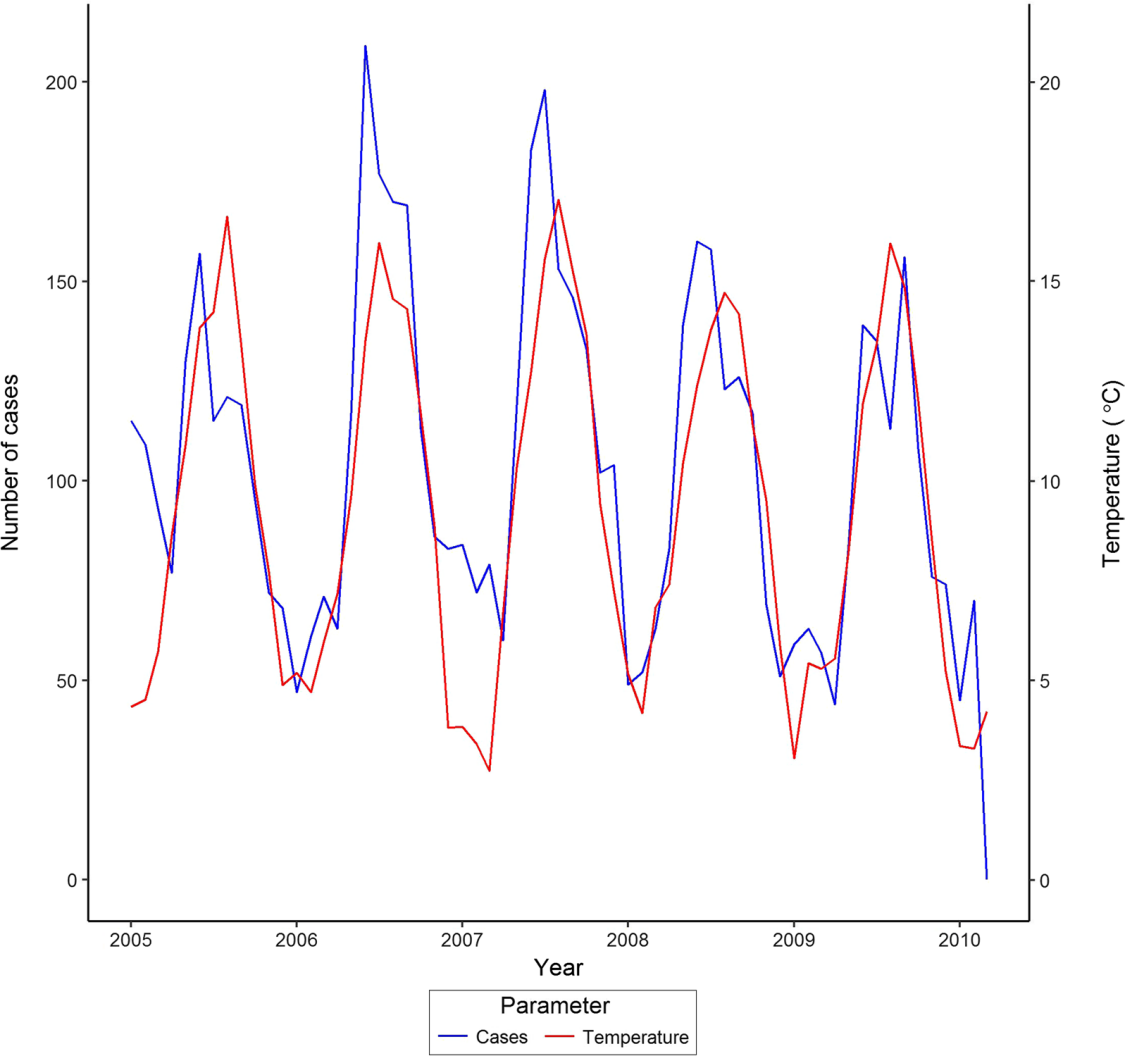
Monthly recorded cases of *Campylobacter* in NE England 2005 to 2009 in relation to temperature

The number of reported cases was highly seasonal rising to a peak in early summer (June) each year and closely matched the seasonality in temperature and rainfall (Fig. 2). The seasonality was well-described with harmonic regression models which were significant for *Campylobacter* cases (t = − 7.448, P < 0.001 and t = − 7.436, P < 0.001 for cosine and sine variables of time with a 365 day period) and monthly mean temperature (t = − 18.710, P < 0.001; t = − 25.300, P < 0.001). There was evidence for periodicity in the rainfall (t = 3.634, P < 0.001 for cosine variable). We used the residuals from these models as de-seasonalised measures of each variable to investigate links between variables and disease. De-seasonalised counts of *Campylobacter* cases were not significantly related to de-seasonalised temperature after also adjusting for autocorrelation (t = 0.212, P = 0.230) or rainfall (t = − 0.119, P = 0.906). This suggests that the simple seasonal relationship between monthly number of cases of *Campylobacter* and mean monthly temperature and rainfall is not a true one and was in fact related to other unmeasured seasonally-varying phenomena.

### Impacts of temperature and rainfall on visits to the countryside of NE England

**Fig. 3:**
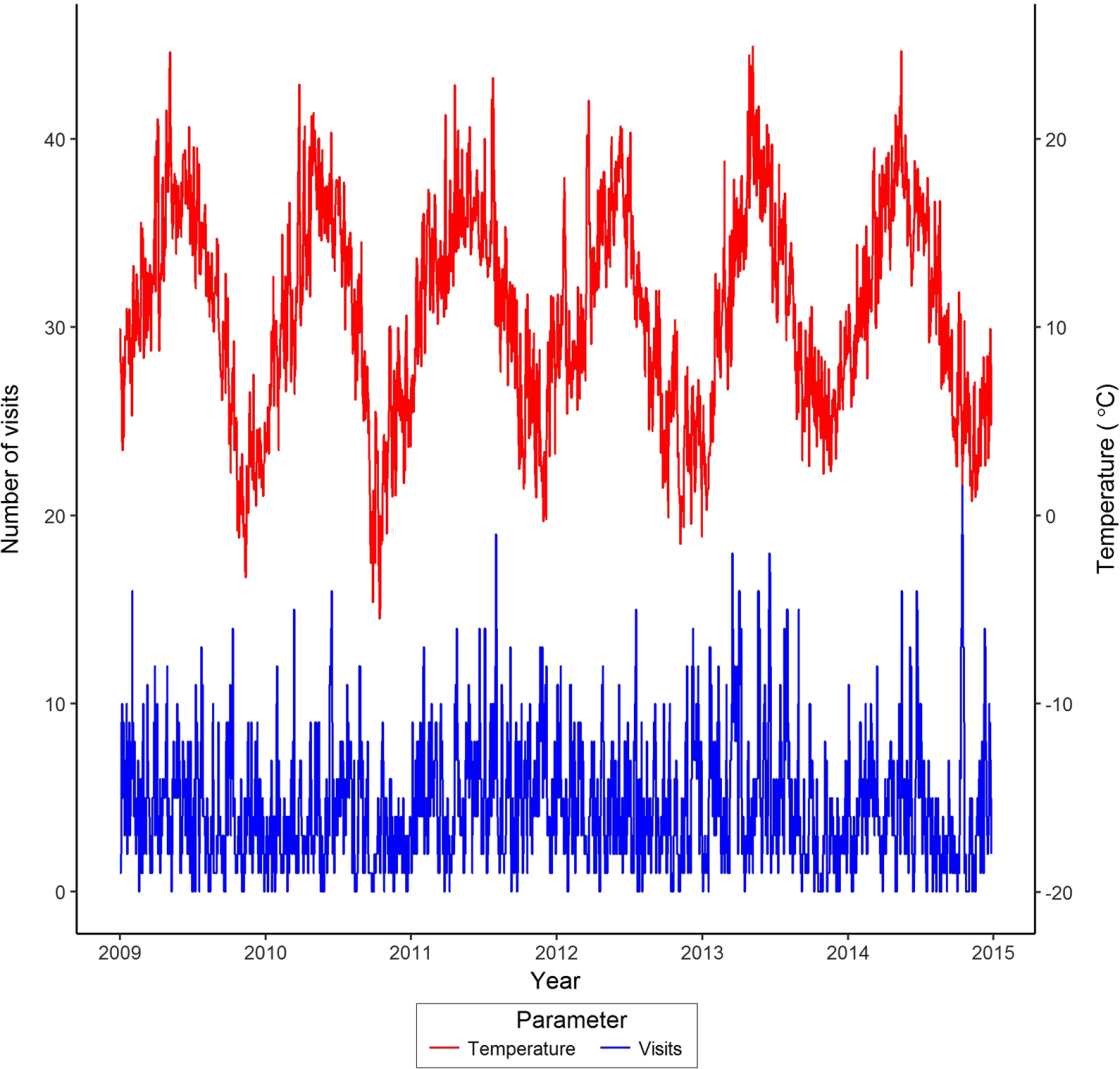
Daily counts of visits to the countryside in the NE England and mean daily temperature, 2009–2015

Total visits to the countryside and daily mean temperature showed seasonal variation across the study period (Fig. 3). Temperature was highly seasonal with the harmonic regression for temperature significant (t = − 65.950, P < 0.001 and t = 45.830, P < 0.001 for cosine and sine variables). There was evidence of seasonal variation in the rainfall (t = − 5.266, P < 0.001 for cosine variable). The log-transformed count of visits to the countryside per day showed marked annual (t = − 4.157, P < 0.001; t = 5.328, P < 0.001) and weekly (t = − 3.220, P = 0.001; t = 5.736, P < 0.001) periodicities, reflecting the seasonal weather and periodicity associated with the working week. There was a significant linear relationship between the de-seasonalised visits and that for temperature and rainfall data (t = 3.665, P < 0.001 and t = − 2.187, P = 0.029 for temperature and rainfall respectively). This suggests that in contrast with the occurrence of cases of disease, weather variables were important drivers of people visiting the countryside. Furthermore, there was a significant relationship between probability of an individual undertaking a visit to the countryside and weather, socio-economics status and age. GEE Wald test statistics (W) indicated that visits to the countryside were positively associated with increased temperature (W = 16.343, P < 0.001), weekends (Saturday: W = 53.370, P < 0.001; Sunday: W = 107.679, P < 0.001), tending to increase with age (W = 22.691, P < 0.001) and higher socio-economic class (W = 47.283, P < 0.001).

### Impact of temperature and rainfall on Internet queries for barbecue charcoal

**Fig. 4:**
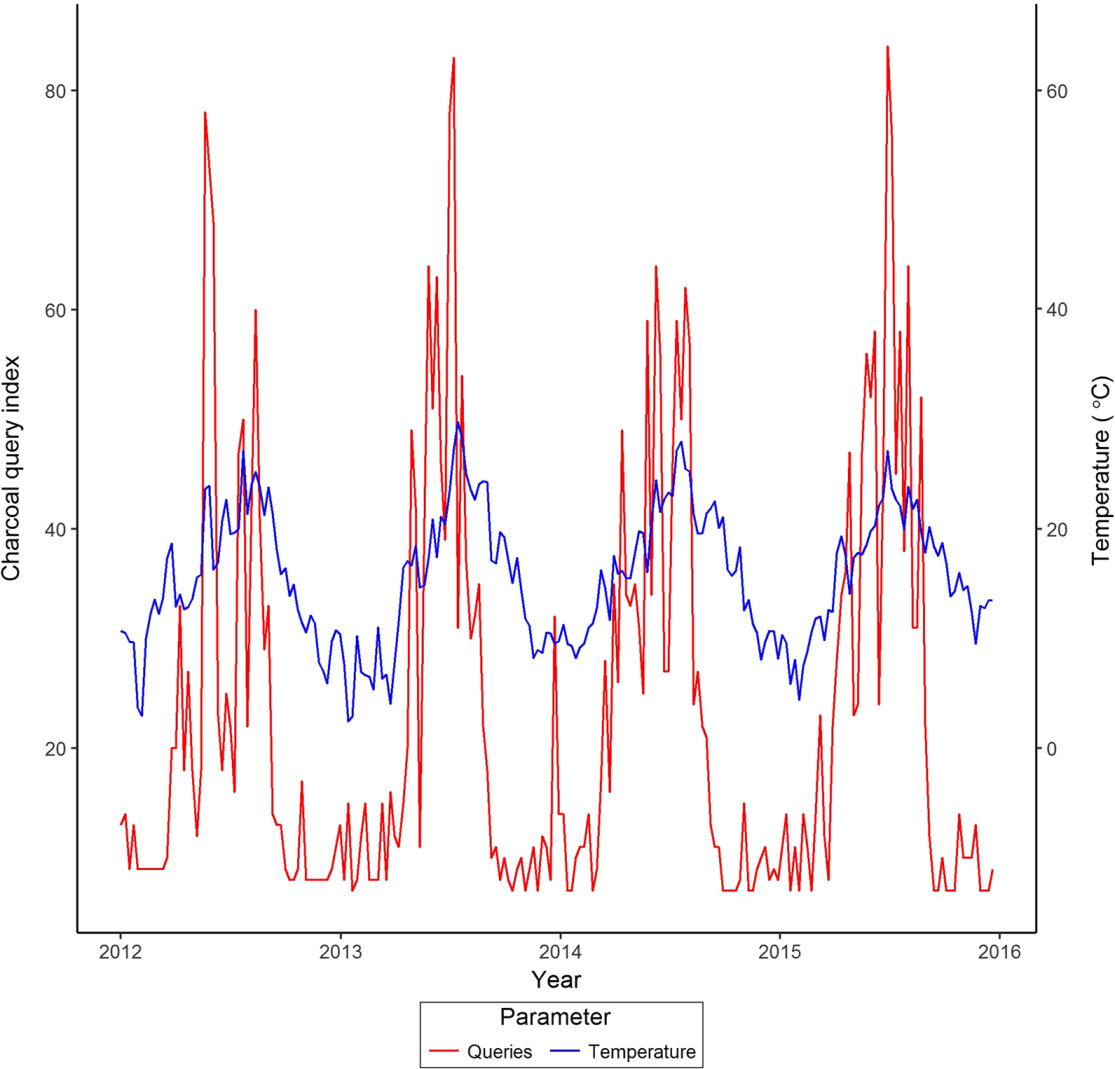
Proportional of queries (index 0 to 100) relating to purchase of barbecue charcoal 2012–2015, and mean monthly temperature

Web queries for barbecue charcoal for England over the study period were used as a surrogate for pursuit of barbecue activities. Queries for information on barbecue charcoal material were highly seasonal (Fig. 4) with significant harmonic regression coefficients (sine t = − 2.606, P = 0.010; cosine t = 2.457, P = 0.015). De-seasonalised query data were significantly related to temperature and rainfall in the week of the queries, suggesting that queries were related to weather rather than other unmeasured seasonal trends. De-seasonalised queries were positively associated with maximum weekly temperature (t = 11.014, P < 0.001) but were negatively associated with the minimum average weekly temperature (t = − 3.626, P < 0.001). This also suggests that, in contrast with the patterns of disease (and perhaps not surprisingly) interest in barbecue charcoal was driven by weather.

### Impact of temperature and rainfall on sales of chicken products

There was a seasonal pattern to the sales of raw chicken products and the harmonic regression for chicken consumption was significant (cosine t = 16.300, P < 0.001; sine t = 15.560, P < 0.001). However, after subsequent de-seasonalising the relationships between chicken sales and temperature and rainfall were not significant (t = − 0.903, P = 0.368 and t = 0.897, P = 0.372, respectively). This suggests that temperature and rainfall were not drivers of chicken purchases.

### Impact of chicken product sales on *Campylobacter* cases

There were no significant relationships between de-seasonalised sales of all raw chicken or barbecue chicken products, and the equivalent de-seasonalised *Campylobacter* cases (t = 0.070, P = 0.945 and t = 1.222, P = 0.234, respectively). This suggests that sales of both all raw chicken and raw “barbecue” chicken alone did not have a direct effect on the numbers of *Campylobacter* cases.

### Impact of monthly total of countryside visits on *Campylobacter* cases

There were no significant relationships between de-seasonalised total monthly visits to the countryside and *Campylobacter* cases (t = − 0.541, P = 0.59). This suggests that monthly visits to the countryside had little influence on numbers of cases.

In summary, the time series analyses suggest that weather appeared to influence visits to the countryside and also the pursuit of barbecues, but was not itself a driver of cases of disease. However the number of cases was associated with our measure of barbecue activity and hence indirectly with weather.

### Results of the IB model

**Fig. 5:**
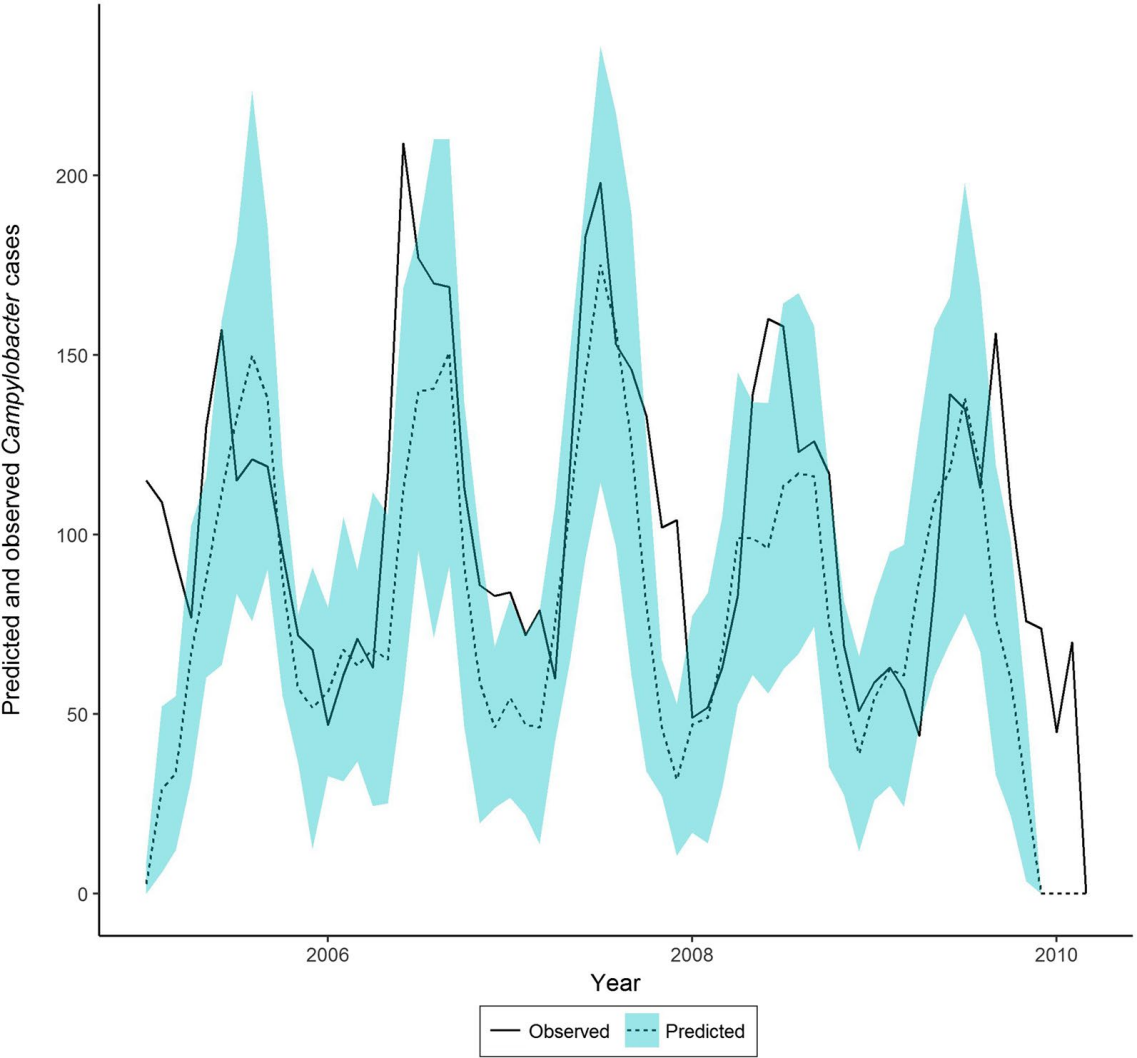
Predicted number of *Campylobacter* cases (rescaled by ×7; see text) in NE England (± sd) attributed to chicken strains 2005 to 2009 and the observed number of cases over the same period

The predicted number of *Campylobacter* cases from the IB model, using weather and socio-demographic data as inputs, followed a cyclic pattern, with cases lowest in winter but rising to a peak in early summer. The observed numbers of cases fitted reasonably well within the 95% confidence intervals for our model predictions. There was a significant positive correlation between the mean numbers of observed and predicted cases per month for NE England over the study period. Mean number of predicted and observed cases per month were compared using generalized linear models, and predictions were significantly related to observations (r = 0.728; t = 8.210, P < 0.001). The regression coefficient was 6.12 (95% CI 4.95 to 8.01); the model over-predicting cases by a factor of 6.12. When the observed data were scaled by a multiplier of seven the match between the predicted and observed cases is clear (Fig. 5). The predicted proportion of *Campylobacter* cases derived from chicken (mean 88.1%, SD 25.9) declined slightly in winter when other strains formed a greater proportion of predicted cases.

### Interventions to mitigate against disease

We altered immunity, daily temperature and rainfall (which affect both barbecue activity and visits to the countryside), probability of under-cooking chicken, and the effectiveness of cooking/reduced cfu load on chicken, and re-ran the IB model to predict number of cases of disease. All interventions significantly reduced the predicted total number of *Campylobacter* cases, but the effectiveness of the interventions differed greatly. Extending the duration of immunity through vaccination of the population had the largest effect on level of disease (t = 56.072, P < 0.001), explaining more than 95% of the variation in predicted number of cases relative to the other interventions. Extending immunity from 20 days to 1 year reduced predicted number of cases by an order of magnitude (95% CI 2412–2414 to 203–309 per 50,000 person-years). There were lower impacts from changes in daily temperature (t = 6.801, P < 0.001) and rainfall (t = 9.538, P < 0.001) which would affect visits to the country as well as the adoption of barbecue activity. Educational interventions to change the probability of under-cooking (t = − 5.963, P < 0.001) and the fold-reduction in *Campylobacter* dose on raw meat before cooking or the effectiveness of the cooking process (t = − 5.540, P < 0.001) had significant effects, but their relative contribution to overall number of cases was small.

## Discussion

To our knowledge our research is the first interdisciplinary study that integrates different and disparate human risk-behaviours, with immunology, demography of the at-risk population, sources of contamination, and weather to predict disease. The models suggest that behaviours driven by weather that lead to consumption of barbecued chicken, and to a lesser extent visits to the countryside, lead to exposure and disease. More importantly, they indicate that consideration of the immune-dynamics of the host–pathogen interaction is necessary to understand the relative role of different exposure pathways to disease.

There are obvious limitations of the modelling. Data were derived from different studies with overlapping time periods. We hypothesised that the processes investigated were both causal and also consistent through time. We cannot assess the impacts of these assumptions on the model formally, but note that the patterns of disease in the UK are predictably consistent year-on-year [17]. We did not model all processes identified as risk factors. We excluded exposure at non-domestic food establishments [32] and cases associated with foreign travel [10, 33, 34]. Strachan et al. [10] suggested national and international travel accounted for 18% and 17% of cases respectively. We note that both of these risk behaviours are likely to be seasonal in themselves. It is difficult to quantify the contribution of cases arising from travel because of poor ascertainment. However, travel in its own right is unlikely to be a mechanism leading to disease, but rather it could lead to changes in human behaviours or in exposure to new strains or both. We also did not model variation in immune response to different *Campylobacter* strains, treating all as homologous in their impacts on development of disease. However, in reality, *C. jejuni* exhibits significant genetic diversity [35]. Furthermore, recent evidence shows that *C. jejuni* undergoes transcriptional and genetic adaptation during human infection [36].

Our analyses of countryside visits and barbecue behaviour suggested that there were significant relationships between both activities and the weather immediately prior to adoption of the behaviour. Thus, there is a mismatch in the time scales of recording of disease and the risk behaviours that lead to exposure to the pathogen. The time-series analyses suggest our proxy for barbecue activity and visits to the countryside were directly related to temperature and rainfall. The former activity has been cited as a risk factor for disease [6, 7, 37] but our results indicate that this risk factor for exposure to the pathogen was mediated by weather. Thus, the seasonal pattern in human *Campylobacter* cases in NE England is probably not directly influenced by weather, but rather by an indirect effect through changing the human behaviours that lead to exposure. The IB model operated at a more short-term timescale than the time-series analyses and allowed for variation in reporting and case ascertainment specifically. The IB modelling results therefore provide more insights into the disease mechanisms than the time-series analyses and allows more scope in an assessing potential intervention strategies. In effect the model predicted population-level patterns of disease based on simulating human behaviour and exposure events for individuals on a daily basis. This more fine-scale modelling showed that weather-driven variations in barbecue activity, countryside visits and domestic cooking provided a reasonable explanation for the broad pattern of observed monthly cases of disease.

The UK-based IID2 study [38] concluded that only around one in seven people with *Campylobacter* symptoms sought medical help. Our model predicted an approximately six-fold difference between predicted infections and observed cases, which whilst possibly fortuitous may reflect this under-reporting to health services. In addition, we predicted that 88% of cases were from strains associated with chicken, similar to findings of Kramer et al. [39] although this is higher than the 40% to 50% reported elsewhere [32]. These results suggest that there is a smaller role for countryside exposure in causing disease in this population, which matches the conclusion we drew from the time-series analyses where it was not a significant predictor at all. Whilst we have outlined the limitations to our model, it should also be stressed that the epidemiological processes that we have omitted or over-simplified could all be readily incorporated with suitable data. The model generates confidence intervals on predictions, which give it inferential power. In addition, notwithstanding social-demographic features of the population which might predispose UK citizens to particular risk behaviours, this modelling approach could be extended to any country where equivalent risk behaviour, consumer and climate data exist.

Our results indicate that the dynamics of a person’s immune response after exposure affect the cyclic pattern of disease in the population and the overall burden of disease. Vaccination to extend short-term immunity was the most important factor determining number of cases. However, the modelled interaction between host and pathogen was probably over-simplistic. Resistance to *Campylobacter* infection is assumed to change with age [40]. This could reflect progressive acquisition of immunity from repeated exposure. In effect, the pool of strains that could initiate disease might decline with repeated exposure. Strains can also have an homologous effect on the immune system, with exposure to one strain leading to immunity to others [41], and protection from subsequent illness [16, 42]. Analyses of strains causing illness in Scotland [43] showed that rare strains appeared more frequently in older patients. However, a small proportion of individuals can shed *Campylobacter* without showing disease [44] or people may have symptoms not sufficiently severe to make them seek medical attention. There has been considerable effort to develop vaccines against *Campylobacter* particularly for livestock [45] and the immunological evidence from animal models suggests that repeated vaccination can lead to medium-term immunity (> 26 weeks).

Vaccines research has mainly focussed on identifying target antigens, particularly proteins and polysaccharides [46]. A conjugate vaccine for enterotoxic bacteria including *Campylobacter* has been shown to lead to functional antibodies to disease in mice [47]. However, developing a vaccine for humans is more complicated because of the poor understanding of the underlying immunology and the potential for interactions with post-infection immunological syndromes like Guillain Barré syndrome [45]. There is also the problem of development costs. It has been estimated that development to the point of drug approval would cost $2.8–3.7 billion [48, 49]. However, the huge expense of vaccine development has to be considered in the context of the cost of the disease burden, which annually in the EU alone has been estimated as of the same order as that of the cost for developing vaccines (€2.4 billion~ €2.7 billion). Equivalent analyses of the cost effectiveness of behavioural interventions to mitigate food-borne disease have been less frequent. One study in the US, with a budget of $300 K, led to a program in which 14,062 people participated with a reduction in disease risk of 12.8% [49]. The benefits of this level of prevention were considered sufficient to outweigh the costs. However, the practicality of behavioural interventions at anything other than the small scale probably means they are impractical given the sizeable disease burden and the lack of efficacy suggested by our analyses.

## Conclusion

This is the first inter-disciplinary study to integrate environment, risk behaviour, socio-demographics and immunology to model infectious disease and identify pathways to mitigation. We conclude that vaccination is likely to be the best route for intervening against campylobacteriosis despite the technical problems associated with understanding both the underlying human immunology and genetic variation in the pathogen, and the likely cost of vaccine development.

## Appendix

See Table 1.

## Abbreviations

GEE: generalised estimating equations
IB: individual-based
MENE: Monitoring of Engagement with the Natural Environment
CFU: colony forming units
